# Attention

**Published:** 1889-08

**Authors:** 


ATTENTION.—To the Medical Profession.—In the especial interest of Doctors, and in the name of economy and fair dealing, you are earnestly requested to assist in making a test of Doctor's buggies, by furnishing our agent, John G. Reed of 311 Elm St. Cincinnati, Ohio, an accurate statement in detail as to make, style, endurance, professional reputation and price, of the buggy you use. The statement to be brought, with hundreds of others before a competent and fairly chosen board of five Judges to ascertain the best make of Physicians buggies, for the money charged for it.

The profession is to have the benefit through this and 19 other Journals of the information thus gained. Please make statement in detail at once and by so doing, confer a favor upon many of the medical fraternity.

EDITOR.

